# Application of the 2-piece orthodontic C-implant for provisional restoration with laser welded customized coping: a case report

**DOI:** 10.1186/s13005-015-0066-9

**Published:** 2015-03-25

**Authors:** Janghyun Paek, Hyo-Won Ahn, Do-Min Jeong, Jeong-Seok Shim, Seong-Hun Kim, Kyu-Rhim Chung

**Affiliations:** Department of Prosthodontics, School of Dentistry, Kyung Hee University, Seoul, Korea; Department of Orthodontics, School of Dentistry, Kyung Hee University, #1 Hoegi-dong, Dongdaemun-gu, Seoul, 130-701 Republic of Korea; Division of Periodontology, Department of Dentistry, National Medical Center of Korea, Seoul, Korea; Department of Orthodontics, School of Medicine, Ajou University, Suwon, Korea

**Keywords:** Dental implant, Congenital missing, C-implant, Osseointegration, Laser welding

## Abstract

This article presents the application of laser welding technique to fabricate an orthodontic mini-implant provisional restoration in missing area after limited orthodontic treatment. A 15-year-old boy case is presented. Two-piece orthodontic C-implant was placed after regaining space for missing right mandibular central incisor. Due to angular deviation of implant, customized abutment was required. Ready-made head part was milled and lingual part of customized abutment was made with non-precious metal. Two parts then were laser welded (Master 1000, Elettrolaser Italy, Verona, Italy) and indirect lab composite (3 M ESPE Sinfony, St. Paul, MN, USA) was built up. The patient had successful result, confirmed by clinical and radiographic examinations. Before the patient is ready to get a permanent restoration later on, this provisional restoration will be used. This case shows that a two-piece orthodontic C-implant system can be used to maintain small edentulous space after orthodontic treatment.

## Background

Space management is one of important issue during retention after orthodontic treatment. Although adequate space is prepared for definitive implants, some patients still want to postpone the installation of the dental implant due to several reasons such as high cost, difficulty in schedule adjustment, or residual alveolar growth in adolescent patients [1] and so on. In that situation, orthodontists usually deliver removable retainers incorporating the missing teeth. However full time wearing of the retainer requires remarkable compliance and if the patients’ cooperation is not satisfactory, the obtained space is easy to be lost.

A single-tooth mini-implant or small diameter dental implants have been reported to be a viable option as an abutment for a crown restoration with a satisfactory long-term period [2]. The C-implant (Cimplant Co., Seoul, Korea) is a 2-piece orthodontic mini-implant system and its clinical application as a skeletal anchorage device has been reported [3-5]. The diameter of C-implant is 1.8 mm similar with other orthodontic mini-implants however, it has SLA surface and the threads with a smooth cutting edge which provide osseointegration like the conventional prosthetic implant. Therefore, the C-implant can be regarded as a miniature of dental implant which has good stability.

If the stability of provisional restoration using the 2-piece orthodontic C-implant is guaranteed, improved esthetics in coronal part is another important issue. The size of C-implant is ideally designed for any small edentulous area, thus facilitates esthetic fabrication of a provisional restoration by indirect build up method. C-implant should be placed following the angle of alveolar crest and buccal angulation of C-implant placement is inevitable in many cases. With buccally placed implant, angular correction should be carried out to fabricate esthetic implant restoration. Laser welding technique is introduced to correct the angle of implant abutment for esthetic restoration while maintaining the frictional fit.

In this report, provisional restoration using the 2-piece orthodontic C-implant as fixed retainer was described which meet both stability and esthetics.

## Case presentation/technique description

**Figure 1:**
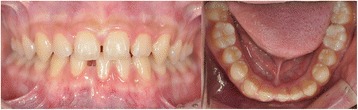
**Pretreatment intraoral photographs of the 15-year-old patient.**

**Figure 2:**
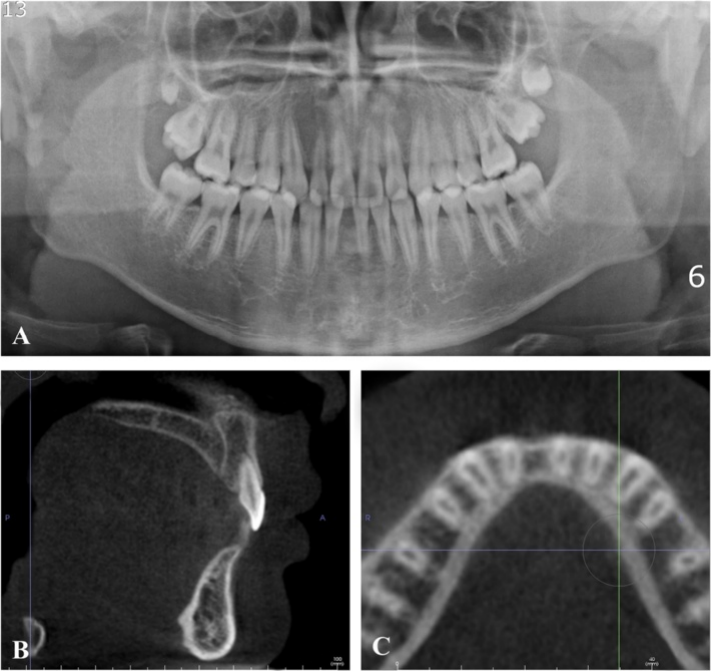
**Pretreatment radiographs. A**. Pretreatment Panoramic radiograph. **B** and **C**. Pretreatment sagittal and axial CBCT images. Because buccal plate of alveolar bone was concave, buccal placement of C-implant was inevitable.

**Figure 3:**
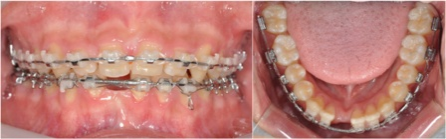
**Three months after treatment.** Anterior spacing was closed and space for implant was gained in right mandibular central incisor area.

**Figure 4:**
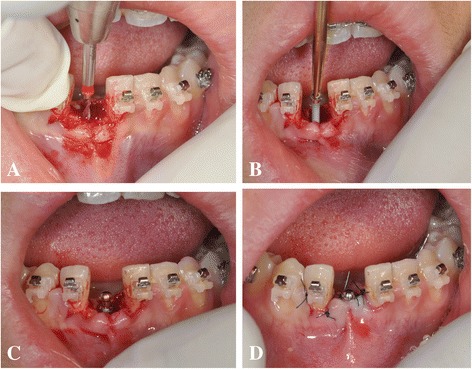
**C-implant placement procedure for anterior mandible (open method). A**. Flap opening and guide drilling (1.5 mm in diameter) after local anesthesia. **B**. Screw part placement using manual driver. **C**. Head part adaptation for healing cap. **D**. Suture.

**Figure 5:**
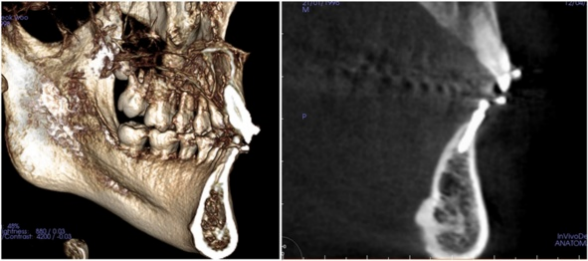
**Post treatment CBCT view.** Because buccal plate of alveolar bone was concave, buccal placement of C-implant was inevitable.

A 15-year-old boy presented with missing right mandibular central incisor and missing space in anterior dentition (Figures 1 and 2). The treatment goal was to close the space and restore the missing tooth. Treatment objectives included reestablishing the original space of the mandibular right central incisor by uprighting the mandibular lateral incisor, minimizing the involvement of the posterior dentition. After 6 months of active orthodontic treatment, however, the size of the regained space was insufficient for placement of a conventional diameter dental implant (Figure 3). Therefore, C-implant (length 10.5 mm, diameter 1.8 mm) was placed in right mandibular central incisor area. The body part of the mini-implant was placed at the crest of the alveolar bone of the mandibular right central incisor following the angle of residual alveolar bone (Figure 4). Posttreatment CT was taken to verify the placement (Figure 5). The 2-mm long head part of the mini-implant assembly was attached to the body and kept in place to ensure gingival patency above the mini-implant body for the fabrication of temporary restoration.

**Figure 6:**
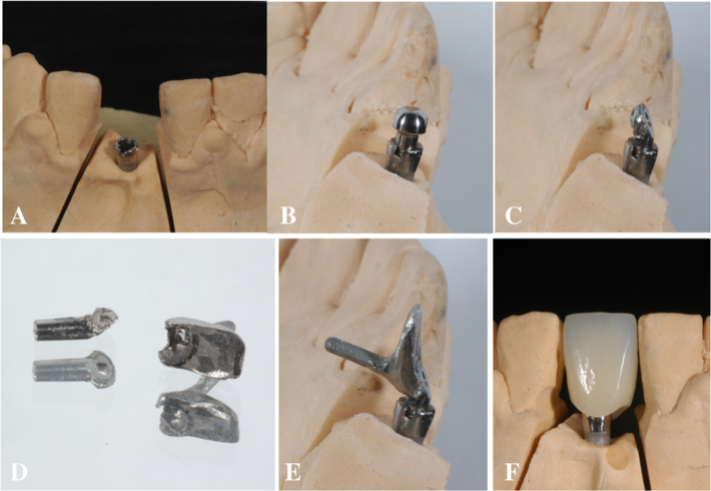
**Laboratory procedures. A** and **B**. Master cast and ready-made head part of the C-implant, **C**. Head part was milled to correct the angle of C-implant, **D**. Lingual part of the customized abutment was fabricated in lost-wax technique, **E**. Ready-made head part of the C-implant was laser welded with customized lingual part of the abutment. **F**. Indirect lab composite (3M ESPE Sinfony) was built up.

The C-implant body connects with its head part by a friction-grip joint. Three lengths of the head part are available (1, 2, and 3 mm) and the size of the head part is determined based on the gingival depth at the alveolar crest. Two millimeters long ready-made head part was connected to the body and used as an impression coping. Pick up impression was taken and C-implant body was connected as an analog. Then, master cast is made with soft tissue cast (Figure 6A and B). In the master cast, 2-mm long ready-made head part was milled to correct the angle of C-implant. To fabricate the ideal contour of the customized abutment, nonprecious metal was cast for the lingual part in conventional lost-wax technique (Figure 6C and D). Milled head part of C-implant and customized lingual part were then laser welded using a dental laser-welding machine (Master 1000, Elettrolaser Italy, Verona, Italy) under argon gas shielding (Figure 6E). The device was with these parameters: wavelength 1064 nm, spot diameter 0.2/2.0 mm, peak power 5 kW, energy 0.1/100 J, pulse frequency 0.5/30 Hz, impulse time 0.1/20 ms. Laser wire NP Co/Cr was used by one-point welding technique.

Indirect lab composite (3M ESPE Sinfony, St. Paul, MN, USA) was then built up and customized staining was done to match the shade (Figure 6F). The final crown was slightly relieved from the occlusion to prevent overload of occlusal force since there is no long-term data reporting the strength of laser welding.

**Figure 7:**
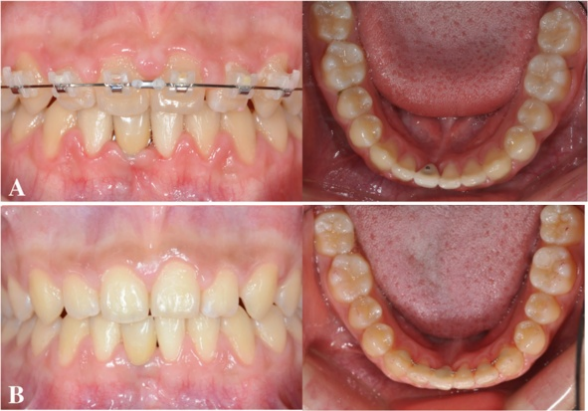
**Posttreatment and retention records. A**. Posttreatment intraoral photographs after ceramic crown cementation. **B**. 1 year after retention.

The posttreatment intraoral photographs of the missing mandibular right central incisor area show normal healing of the alveolar bone around the mini-implant (Figure 7A). One year after debonding, the restoration was well maintained, and the width and contour of attached gingiva adjacent restoration was more favorable (Figure 7B).

## Discussion

Considering the patient’s young age and anticipated growth potential, a definitive implant restoration for the missing tooth should be postponed until the general growth is complete. The residual alveolar bone, however, was expected to resorb if a conventional fixed or removable orthodontic retainer was used to maintain the space, since these appliances do not have bone-preserving properties. Therefore, an orthodontic mini-implant interim restoration is advantageous to prevent alveolar bone resorption and satisfy esthetic concerns and until general growth is completed in the next several years. The patient was informed about the need to have a final implant restoration upon completion of growth, since the gingival level of the alveolar crest of interim mini-implant restoration is expected to be more apical than adjacent teeth in several years [6]. Another advantage of making interim restoration with orthodontic mini-implant is that the orthodontic mini-implant cast-crown restoration is significantly cheaper than the prosthesis on commercially available small diameter dental implants because the orthodontic mini-implant system does not require additional system-specific surgical kits, which increase the base fee of dental implant restoration. Not only surgical kit, but prosthetic components such as impression coping and implant analog are not required. Moreover, the C-implant system used in this report is two-piece system consisted of the body part and head part. As explained above, because the head part can be modified as abutment, there is no need to purchase another abutment. In this specific case, the size of head part provided maximum space for laser welding which enabled strong connection between head part and abutment. With previously mentioned advantages, the orthodontic mini-implant is gaining popularity as a viable option as provisional restoration in adolescent patients.

Laser technology is the most efficient method when applying thermal energy to small areas and it is one of the best fusion welding techniques for dissimilar metals. Therefore, laser welding under argon shielding is a useful method in joining titanium and its alloys, overcoming the high reactivity and strong affinity of titanium with gasses such as oxygen, hydrogen and nitrogen at high temperatures [7-10].

There are many other space maintaining options in partially edentulous adolescent patients. Non-invasive adhesion fixed partially denture and interim removable partial denture are widely used in many cases. However, the former cannot prevent alveolar bone resorption and it cannot be used when there is not enough space for the adhesion wing [11-13]. The latter has esthetic problem due to its clasp and compliance issues especially with adolescent patients. As described in this report, provisional restoration with C-implant is mainly for space maintaining, therefore, the focus is on esthetics instead of function. It will help maintain the bone level of the alveolar ridge and reduce the need for future bone graft. As the surface of C-implant is sandblasted and acid-etched, the long-term use of the C-implant for orthodontic anchorage has proven to be stable under multidirectional forces [14]. However, there is no study reporting the long-term stability of the orthodontic mini-implant interim crown. Further investigation on the long-term stability will be needed. In this report, one-year follow up was performed and the result was satisfactory.

## Conclusions

A 2-piece orthodontic C-implant system can serve as an excellent treatment option to retain edentulous space after orthodontic treatment until the future definitive restoration can be made.

## Consent

Written informed consent was obtained from patient and parents for publication of this report and the accompanying images. A copy of the written consent is available for review by the Editor-in-Chief of this journal.
